# CLEAR results, cloudy impact: colchicine's neutral role in ST-segment elevation myocardial infarction

**DOI:** 10.1093/ehjcvp/pvaf011

**Published:** 2025-03-13

**Authors:** Claudio Laudani, Antonio Abbate, Dominick J Angiolillo, Mattia Galli

**Affiliations:** Division of Cardiology, Azienda Ospedaliero-Universitaria Policlinico “Rodolico—San Marco”, University of Catania, Catania 95100, Italy; Division of Cardiology, University of Florida College of Medicine-Jacksonville, Jacksonville, FL 32209, USA; Division of Cardiology and Berne Cardiovascular Research Center, University of Virginia, Charlottesville, VA 22903, USA; Division of Cardiology, University of Florida College of Medicine-Jacksonville, Jacksonville, FL 32209, USA; Department of Medical-Surgical Sciences and Biotechnologies, Sapienza University of Rome, Via XXIV Maggio, 7, 04100, Latina, Italy; Maria Cecilia Hospital, GVM Care & Research, Via Corriera 1, 48033, Cotignola, Italy

The current understanding of atherosclerotic disease development underscores the pivotal role of inflammation in the initiation, progression, and destabilization of arterial plaques. Anti-inflammatory therapy has recently emerged as a promising therapeutic approach for patients with coronary artery disease (CAD), with colchicine being the most extensively studied agent in this context.^1^ Importantly, compared to other strategies such as single or dual antiplatelet therapy (DAPT) or dual-pathway inhibition combining antiplatelet therapy with a low dose of a direct oral anticoagulant, targeting the inflammatory pathway does not have the drawback of increasing the risk of bleeding complications.^2^ To date, 19 clinical trials on colchicine in patients with CAD have been conducted, yielding mixed results regarding its efficacy and safety in cardiovascular prevention (*Figure 1*).^1^ Based on the positive results of the pivotal large-scale low-dose colchicine for secondary prevention of cardiovascular disease (LoDoCo2) and colchicine cardiovascular outcomes trial (COLCOT) trials, colchicine is now recommended for secondary prevention in patients with previous myocardial infarction (MI).^3^ However, the recent Colchicine and Spironolactone in Patients with Myocardial Infarction/SYNERGY Stent (CLEAR SYNERGY) (OASIS 9) trial, the largest trial to date evaluating colchicine in patients with CAD, has cast doubt on its efficacy in the setting of ST-segment elevation MI (STEMI), raising important questions about the role of colchicine in cardiovascular risk reduction.^4^

**Figure 1:**
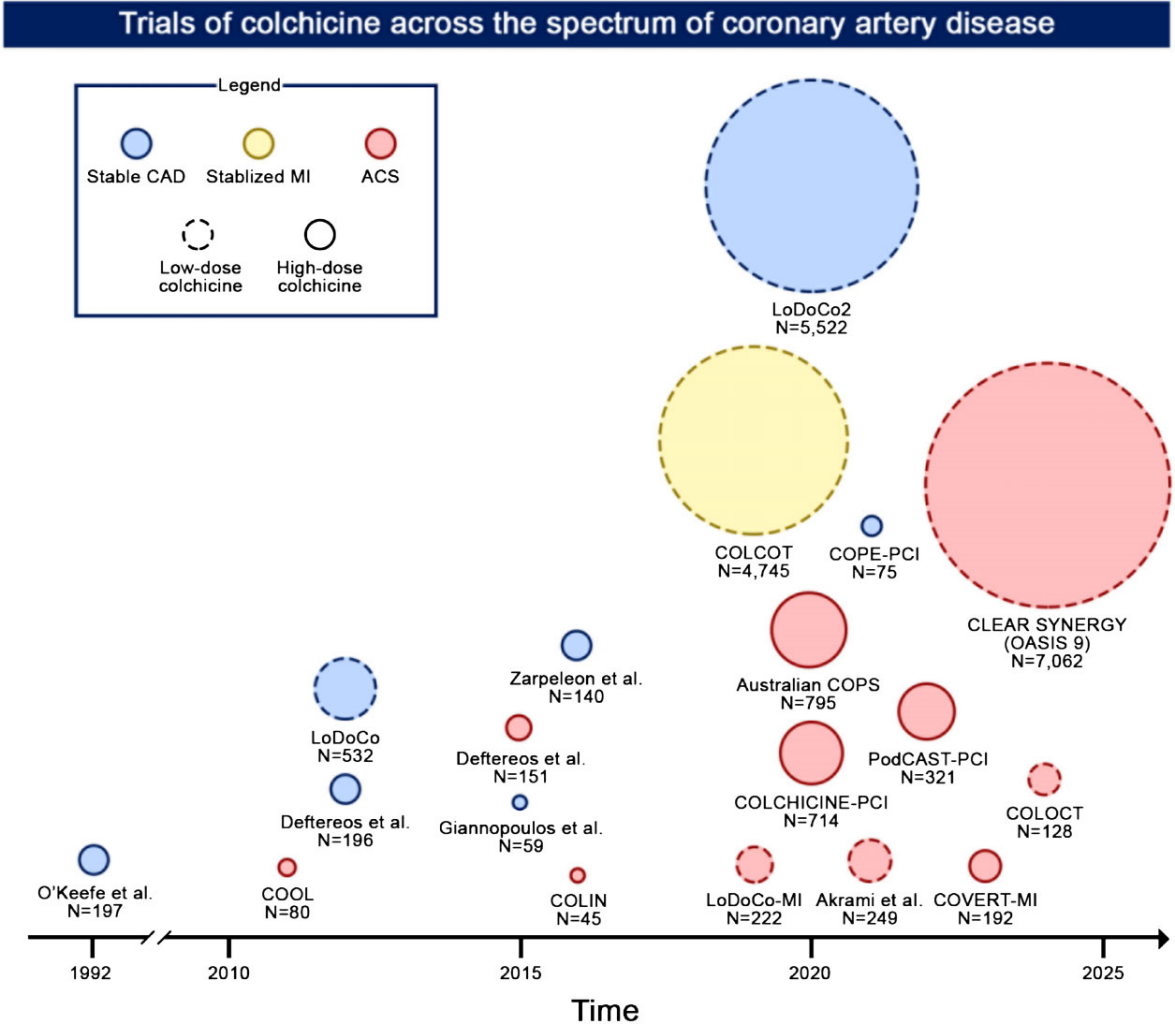
Trials of colchicine across the spectrum of CAD. The present figure illustrates trials of colchicine conducted across the spectrum of CAD over time. Low-dose colchicine refers to 0.5 mg OD, while high-dose colchicine to other dosages. Circles’ dimensions are proportional to the number of patients enrolled. Abbreviations: ACS, acute coronary syndrome; CAD, coronary artery disease; MI, myocardial infarction and OD, once daily.

Specifically, the CLEAR SYNERGY (OASIS 9) was a randomized, double-blind, placebo-controlled trial conducted in 7062 patients with acute MI to either receive colchicine or placebo as soon as possible after percutaneous coronary intervention (mean time 1.6 h). After a median follow-up of 2.98 years, the authors reported overall neutral results for the primary endpoint of major adverse cardiovascular events (MACE), defined as the composite of cardiovascular death, recurrent MI, stroke or ischaemia-driven revascularization (9.1% vs. 9.3%; hazard ratio 0.99; 95% confidence interval 0.85–1.16).^4^ These findings are in contrast with the results of the COLCOT and LoDoCo2 trials, enrolling 4745 and 5522 patients, respectively, that found colchicine to significantly reduce MACE compared to placebo in patients with recent acute coronary syndrome or stable CAD, respectively.^5,6^

The reasons behind these conflicting results are the subject of intense debate. First, the CLEAR SYNERGY (OASIS 9) trial represents the first large trial specifically designed to evaluate the effects of early administration of colchicine in patients with STEMI, while in COLCOT colchicine was given within 30 days after STEMI or non-STEMI (median of 14 days), and the LoDoCo2 trial only focused on patients with stable CAD.^4–6^ These differences suggest that colchicine may be less effective in the acute and sub-acute STEMI settings, characterized by a pronounced inflammatory response, where the initial cytokine surge may overwhelm colchicine's anti-inflammatory effects. However, further evidence is needed to support this hypothesis also in light of the contrasting evidence from small randomized trials testing the effectiveness of colchicine in reducing infarct size in the acute phase of MI patients.^2^

The neutral findings in the CLEAR SYNERGY trial may, at least in part, be attributed to the colchicine dosing regimen used. In fact, patients weighing >70 kg were initially prescribed 0.5 mg twice daily, but a protocol amendment transitioned all patients to 0.5 mg daily after 90 days due to high discontinuation rates. Notably, a subgroup analysis showed a trend toward benefit with the higher dose, raising concerns about the adequacy of the colchicine dose used in the trial.^4^

In addition to timing and dosing, the concomitant therapy administered across trials may explain the conflicting results of randomized trials on CAD patients. The CLEAR SYNERGY trial had the highest proportion of patients on DAPT (98.4%), with ticagrelor and prasugrel as the most common P2Y_12_ inhibitors. Among colchicine's complex and not fully understood mechanisms of action, its inhibition of platelet activation via interference with enzymes involved in cytoskeletal rearrangement may contribute to its observed benefits in CAD patients.^2^ Since these enzymes appear to act downstream of multiple platelet surface receptors, including the P2Y_12_ receptor, it can be hypothesized that colchicine's cardiovascular benefits stemming from its antiplatelet properties may be diminished when DAPT using potent P2Y_12_ inhibitors is used, thereby masking its overall efficacy.^2^ Further efforts should aim to identify patient subgroups most likely to benefit from colchicine, such as those who cannot tolerate antiplatelet agents, those on single antiplatelet therapy regimens, those de-escalated from potent P2Y_12_ inhibitors to clopidogrel or those with specific genetic mutations or ethnic groups affecting clopidogrel response.^7–10^

Finally, the timing of the CLEAR SYNERGY trial, which began before the COVID-19 pandemic and continued with over 50% of patients enrolled during the pandemic, may have significantly influenced outcomes. The COVID-19 pandemic placed immense strain on healthcare systems, complicating the management and follow-up of patients with both acute and chronic conditions and adversely affected the conduct of clinical trials during 2021–2023. When looking at the subgroup analysis prior to COVID-19, there was a trend for a 22% reduction in the incidence of the primary endpoint, which was lost during the pandemic period.

The conflicting outcomes of large trials highlight the need for robust studies to strengthen guidelines on colchicine use and a deeper understanding of its mechanisms, particularly in combination with regimens of antiplatelet therapy with different potency. Identifying patient profiles that benefit from dual targeting of inflammation and platelet pathways could enable personalized treatments and improved outcomes.

## Notes

**Conflict of interest:** A.A. has received consulting fees or honoraria from Kiniksa, MonterosaTx, and Novo Nordisk. D.J.A. declares that he has received consulting fees or honoraria from Abbott, Amgen, Anthos, AstraZeneca, Bayer, Biosensors, Boehringer Ingelheim, Bristol Myers Squibb, Chiesi, CSL Behring, Daiichi-Sankyo, Eli Lilly, Faraday, Haemonetics, Janssen, Merck, Novartis, Novo Nordisk, PhaseBio, PLx Pharma, Pfizer, and Sanofi; D.J.A. also declares that his institution has received research grants from Amgen, AstraZeneca, Bayer, Biosensors, CeloNova, CSL Behring, Daiichi-Sankyo, Eisai, Eli Lilly, Faraday, Gilead, Idorsia, Janssen, Matsutani Chemical Industry Co., Merck, Novartis, and the Scott R. MacKenzie Foundation. The other authors have no conflict of interest to declare.
